# Optimized planting density enhances cotton yield in cotton–peanut intercropping via improved photosynthesis and dry matter partitioning

**DOI:** 10.3389/fpls.2026.1798068

**Published:** 2026-05-07

**Authors:** Shuting Zhang, Xinyue Zhang, Jinbin Wang, Hang Qiao, Guoping Wang, Sumei Wan, Zhengjun Cui, Guodong Chen

**Affiliations:** 1 College of Agriculture, Tarim University, Alar, China; 2 Key Laboratory of Genetic Improvement and Efficient Production for Specialty Crops in Arid Southern, Alar, China; 3 State Key Laboratory of Cotton Bio-Breeding and Integrated Utilization, Anyang, China

**Keywords:** cotton, intercropping, photosynthetic characteristics, planting density, planting pattern, yield

## Abstract

Intercropping and increased planting density are important agronomic strategies to alleviate the conflict between grain and cotton production and to improve land use efficiency. Cotton-peanut intercropping can enhance the land equivalent ratio (LER), and appropriate planting density is critical for achieving high yields. However, the effects of increased density on the photosynthetic characteristics and yield of intercropped cotton in the arid region of southern Xinjiang remains unclear. To address this issue, a field experiment was conducted in southern Xinjiang during 2024–2025 using a split-plot design. The main plots included planting patterns: monocropped cotton (MC), cotton-peanut intercropping IC, and monocropped peanut (MP), while subplots consisted of three planting density (D1: 180, 000 plants·ha^-^¹, D2: 240, 000 plants·ha^-^¹, D3: 300, 000 plants·ha^-^¹). The study systematically investigated the effects of planting density and pattern on cotton photosynthetic characteristics, chlorophyll fluorescence parameters, dry matter accumulation, and yield. The results showed that: (1)The net photosynthetic rate (Pn), transpiration rate (Tr), and stomatal conductance (Gs) initially increased and then decreased during the growth period, with peak values observed at the flowering and boll-setting stage. Under intercropping conditions, the ICD2 treatment exhibited the highest Pn, Gs, and Tr at this stage. In both 2024 and 2025, intercropped cotton under D2 showed 10.1% and 15.6% higher Gs and15.9% and 21.3% higher Fv/Fm, respectively, compared with monocropped cotton at the flowering and boll-setting stage. (2) Dry matter accumulation in all treatments followed a logistic growth model. The onset of rapid accumulation (T1) generally occured earlier in intercropping than in monocropping systems, with ICD3 occurring 10.3 days earlier than MCD3. The ICD2 treatment achieved the highest total dry matter accumulation, exceeding MCD1 by 20.0% in 2024. (3) Cotton yield under monocropping decreased with increasing planting density, whereas under intercropping, yield first increased and then declined, with ICD2 producing the highest seed cotton yield. The land equivalent ratio reached 1.21. (4) Yield was significantly positively correlated with Pn and Fv/Fm at the flowering and boll-setting stages. Principal component analysis further indicated that ICD2 exhibited the best overall performance. In conclusion, cotton–peanut intercropping at a planting density of 240, 000 plants·ha^-1^ (D2) effectively improve cotton photosynthetic capacity, enhances PSII energy conversion efficiency, promotes dry matter accumulation, and increases yield. These findings provide a theoretical basis for optimizing cotton production in the arid regions of southern Xinjiang.

## 1 Introduction

Cotton serves as a vital strategic economic crop in China (Feng et al., 2022). As the largest cotton-producing region in China, Xinjiang accounts for more than 90% of the national cotton outputand plays a critical role in ensuring the security of China industry and maintaining socio-economic stability in border regions (Feng et al., 2024). However, under the constraint of limited arable land resources, the continuous expansion of cotton cultivation inevitably competes with grain and oilseed crops for cropland. This intensifies the land-use conflict between grain and cotton production and poses potential risks to national agricultural supply security (Zhu et al., 2023). Therefore, developing efficient and intensive cropping systems that simultaneously enhance grain and oilseed production while maintaining cotton output capacity is of great strategic importance for sustainable agricultural development and national food security.

Intercropping is an effective approach to alleviating the “grain-cotton land competition” by improving resource use efficiency through complementary ecological niches between crops (Zou et al., 2023). The cotton-peanut intercropping system represents a typical combination of tall and short crops, utilizing differences in morphological structure and physiological function (Chi et al., 2023). Cotton is a tall crop with high nitrogen demand, whereas peanut is a low-growing legume capable of biological nitrogen fixation (Chen et al., 2024). Peanut (*Arachis hypogaea* L.) is an important oilseed and high-protein crop in China and serve as a potential substitute for soybean imports, contributing to national food security. Peanuts are widely cultivated in northern China. However, continuous peanut monoculture increases the risk of soil-borne diseases, such as peanut stem rot and bacterial wilt (Fang et al., 2023).These two crops pronounced significant spatiotemporal and functional niche differentiation in light, heat, water, and nutrients, thereby enhancing overall resource-use efficiency in intercropping systems (Chi et al., 2026; Zhang et al., 2026). Nevertheless, to fully exploit the yield advantage of intercropping systems, optimizing planting density remains a key technical challenge (Zhang et al., 2025). Planting density determines population structure and regulates the distribution and competition for light and thermal resources, thus playing a central role in yield formation and system stability (Du et al., 2021).

Photosynthesis is the fundamental physiological process governing biomass accumulation and yield formation, with more than 90% of cotton yield derived from photosynthates after the flowering and boll-setting stage (Peng et al., 2025). Key indicators of photosynthetic performance include net photosynthetic rate, stomatal conductance, intercellular CO_2_ concentration, and transpiration rate (Pan et al., 2025). Chlorophyll fluorescence parameters, particularly the maximum quantum efficiency of PSII, are sensitive indicators of the functional status of photosynthetic apparatus (Almeida et al., 2025; Kalaji et al., 2016). Planting density directly influences crop light-use efficiency by regulating canopy structure (Raza et al., 2025). An optimal density improves canopy architecture and enhances light interception, whereas excessive density intensifies interplant competition, resulting in shading of lower leaves and reduced photosynthetic capacity (Nie et al., 2026). Based on the cultivation strategy of “high-density, small plants” systems in the drip-irrigated cotton region of Xinjiang and the requirements of mechanical harvesting, three planting densities were established: 180, 000 (D1), 240, 000 (D2), and 300, 000 plants·ha^-1^. Among these, D1 represents the conventional high-yield density used in local mechanized cotton production (control), D2 represents the a moderate densification level, and D3 approached an ultra-high density threshold. This design aims to systematically evaluate the effects of density on the population structure, individual plant development, and yield formation, and to identify the optimal density range and potential yield-limiting mechanisms in the oasis cotton production systems of Xinjiang. Therefore, elucidating how increased planting density regulates the photosynthetic characteristics of intercropped cotton is essential for understanding yield formation mechanisms and optimizing cultivation practices.

Current research on cotton–peanut intercropping has mainly focused on traditional agricultural regions such as the Huang–Huai–Hai Plain, whereas studies on density optimization under this system are still limited in the arid oasis irrigation areas of southern Xinjiang (Bai et al., 2026). Although this region benefits from abundant solar radiation, severe water scarcity and unique ecological conditions impose greater challenges on crop management (Croce et al., 2024). Existing studies primarily emphasize yield advantage, with limited attention to the underlying physiological mechanisms. In particular, how increased planting density regulates photosynthetic processes in cotton and how these physiological responses influence yield components and final lint yield remain poorly understood in cotton–peanut intercropping systems in the arid region of southern Xinjiang.

Therefore, a field experiment was conducted in southern Xinjiang to investigate the effects of different planting densities and intercropping patterns. Photosynthetic and chlorophyll fluorescence parameters were measured at different growth stages, combined with analyses of dry matter accumulation and yield components. The objectives of this study were to: (1) clarify the effects of increased planting density and peanut intercropping on cotton photosynthetic characteristics; (2) elucidate how increased planting density influences light-use efficiency and yield in the cotton–peanut intercropping system; (3) determine the optimal planting density and intercropping pattern. The results will provide a theoretical basis and technical support for efficient cotton prodcution in southern Xinjiang.

## 2 Materials and methods

### 2.1 Overview of the test site

**Figure 1:**
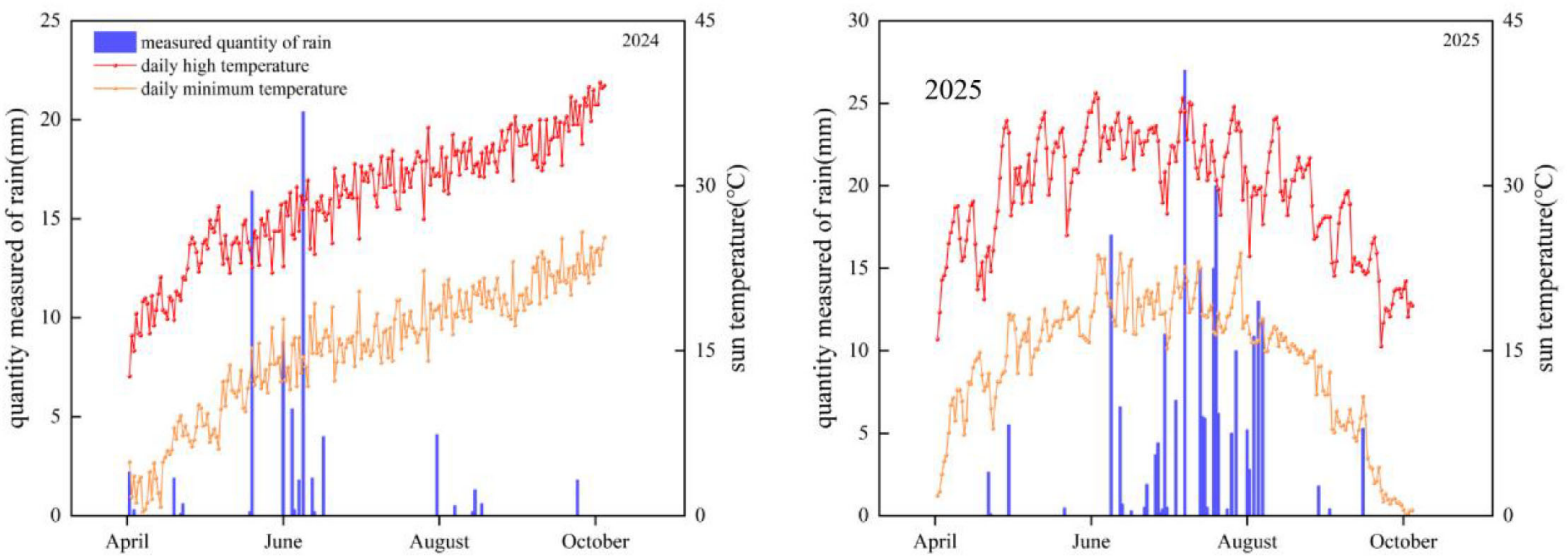
Rainfall, maximum temperature and minimum temperature in 2024-2025.

The experiment was conducted from 2024 to 2025 at the Modern Agricultural Training Base of Industry–Education Integration, Tarim University, located in southern Xinjiang, China (E: 81°4 ‘40.94 “, N: 40°39’ 0.03”; altitude: 1, 100 m). The region is characterized by a typical arid climate, with low precipitation and high evaporation. Based on long-term meteorological data (1982~2025), the annual mean temperature is 12.18 °C, with an average frost-free period of approximately 195 days and annual precipitation of 78 mm. During the experimental years, total precipitation was 70.3 mm in 2024 and 125.8 mm in 2025, while the mean annual temperatures were 11.5 °C and 12.2 °C, respectively (**Figure 1**). The soil in the plow layer (0~20 cm) contained 11.15 g·kg^-1^ organic matter, 0.74 g·kg^-1^ total nitrogen, 37.74 mg·kg^-1^ available phosphorus, and 129.34 mg·kg^-1^ available potassium. A subsurface drip irrigation system commonly used in the region was adopted, with unified water and fertilizer management. The total irrigation quota during the cotton growing season was 4, 500 m³·ha^-1^, while that for peanut was 3, 000 m³·ha^-1^, both applied through staged drip irrigation according to crop demand. For cotton, basal fertilization consisted of organic and compound fertilizers, while top-dressing was applied via drip fertigation. During the growing season, total nutrient inputs were 270 kg·ha^-1^ N, 90 kg·ha^-1^ P, and 150 kg·ha^-1^ K. For peanut, basal fertilization included organic and specialized fertilizers, with top-dressing supplying 80 kg·ha^-1^ N, 120 kg·ha^-1^ P, and 140 kg·ha^-1^ K.

### 2.2 Experimental design

**Figure 2:**
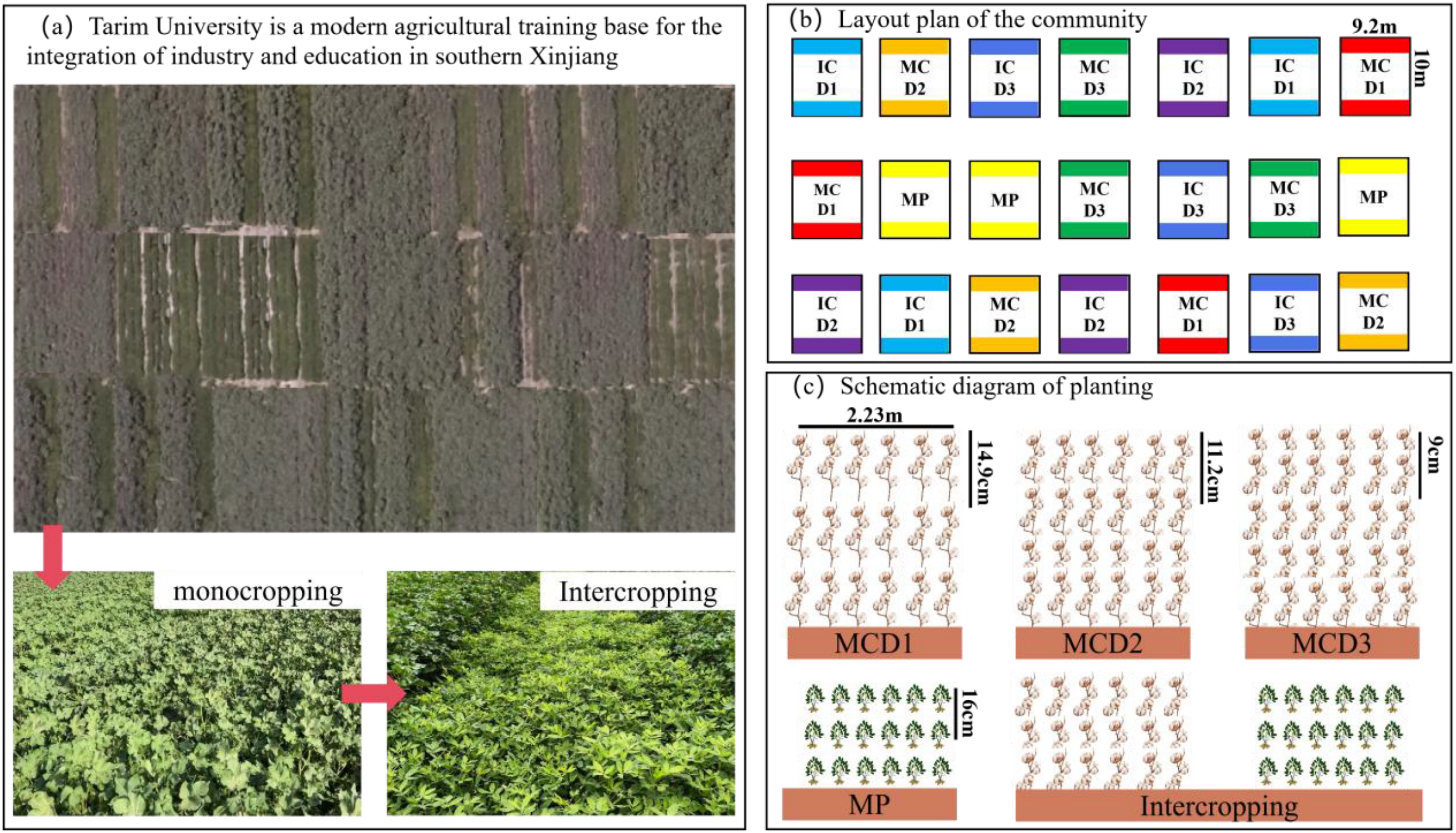
Schematic layout of the residential area.

This experiment employed a split plot design, with planting pattern as the main plot factor (MC: cotton monoculture, IC: cotton-peanut intercropping, MP: peanut monoculture) and planting density as the subplot factor (D1: 180, 000 plants·ha^-1^, D2: 240, 000 plants·ha^-1^, D3: 300, 000 plants·ha^-1^). A total of seven treatments were established, each with three replicates, resulting in 21 experimental plots (**Table 1**). Each plot covered an area of 92 m^2^ (9.2 m × 10 m) and was arranged in an east–west orientation. The cotton cultivar used was Tahe No.2, characterized by a compact plant type, medium plant height, early maturity, and suitability for dense planting. The peanut cultivar was Huayu No.25, with a growth duration of approximately 129 days, upright growth habit, sparse branching, continuous flowering, and clustered pod formation. Both cotton and peanuts were planted under plastic mulch with six rows per film, and each plot contained four mulch films (**Figure 2**).In the intercropping system, cotton and peanut were arranged alternately with one film spacing between peanut rows. Both crops were sown as single seeds. Under D1, D2, and D3 densities, cotton plant spacing within rows was 14.9 cm, 11.2 cm, and 9 cm, respectively, while peanut spacing was maintained at 16 cm.

**Table 1:** Cotton-peanut planting pattern and density treatment design.

planting pattern	Cotton density (10, 000 plants·ha^-1^)	Process ID
Single-crop cotton (MC)	18(D1)	MCD1
	24(D2)	MCD2
	30(D3)	MCD3
Cotton and Peanut Intercropping (IC)	18(D1)	ICD1
	24(D2)	ICD2
	30(D3)	ICD3
Peanut monoculture (MP)	16	MP

### 2.3 Measurement items and methods

#### 2.3.1 Photosynthetic data measurement

Photosynthetic parameters were measured using a LI-6800 portable photosynthesis system (LI-COR Inc., NE, USA). Three representative plants were selected from each plot at the seedling, bud, flowering and boll-setting, and boll-opening stages.The measured parameters included net photosynthetic rate (Pn), transpiration rate (Tr), stomatal conductance (Gs), and intercellular CO_2_ concentration (Ci) (Yao et al., 2016). Each leaf was measured three times, and the average value was used for analysis.

#### 2.3.2 Chlorophyll fluorescence measurement

Chlorophyll fluorescence parameters were measured using a portable fluorometer (Yaxin-1105) at the seedling stage, bud stage, flower-bell stage, and floret stage (Baker and Oxborough, 2004). Leaves were dark-adapted for 30 min prior to measurement, and the maximum quantum efficiency of PSII (Fv/Fm) was recorded.

#### 2.3.3 Measurement of cotton dry matter

In both 2024 and 2025, cotton plants were sampled at 30, 50, 68, 80, 100, and 115 days after emergence. Three representative plants were collected from each plot. Samples were separated into different organs, oven-heated at 105 °C for 30 min to deactivate enzymatic activity, and then dried at 85 °C to constant weight before weighing (Abudurezike et al., 2025).

#### 2.3.4 Measurement of dry matter accumulation

The dry matter content of cotton was fitted using a logistic curve. The Equation 1, Y represents dry matter accumulation (kg·ha^-1^), K denotes the theoretical maximum dry matter accumulation (kg·ha^-1^), and t indicates the number of growth days (d). By calculating the first, second, and third derivatives of Equation, we determined the initial phase (t_1_) and terminal phase (t_2_) of the rapid dry matter accumulation period, along with the maximum accumulation rate (V_m_) and its occurrence time (t_m_) (Dong et al., 2025).

(1) $$\text{Y}=\frac{\text{A}}{1+{\text{be}}^{-\text{kt}}}$$

(2) $$t_{1}=\frac{1}{b}ln\frac{2+\sqrt{3}}{a}$$

(3) $$t_{2}=\frac{1}{b}ln\frac{2-\sqrt{3}}{a}$$

(4) $$t_{m}=-\frac{ln\;a}{b}$$

(5) $$V_{m}=\frac{bK}{4}$$

#### 2.3.5 Yield measurement

Cotton and peanut yields were determined at the boll-opening stage (cotton) and maturity stage (peanut). In each plot, a representative harvest area of 6.67 m^2^ was selected, and all plants within this area were harvested to determine actual yield (Wang et al., 2022).

Density Contribution Rate (RCTD).

(6) $$RCT_{D}=\frac{Y_{D2}-Y_{D1}}{Y_{D1}}$$

where Y_D2_ is the yield at high density within the same cropping pattern, and Y_D1_ represents the yield under lower planting density within the same cropping pattern (Yang et al., 2016).

#### 2.3.6 Land equivalent ratio

The land equivalent ratio (LER) was calculated as:

(7) $$LER=\frac{Y_{IC}}{Y_{MC}}\times 0.5+\frac{Y_{IP}}{Y_{MP}}\times 0.5$$

Where Y_IC_ and Y_MC_ represent the intercropping yield and single-cropping yield of cotton, respectively; Y_IP_ and Y_MP_ represent the yields of peanut under intercropping and monocropping (Wang et al., 2022).

### 2.4 Data analysis

Statistical analyses were performed using IBM SPSS Statistics 27 (IBM Corp., Armonk, NY, USA). One-way analysis of variance (ANOVA) was used to evaluate differences among treatments. Mean comparisons were conducted using the least significant difference (LSD) test and Duncan’s multiple range test at a significance level of *P* < 0.05. Pearson correlation analysis was applied to examine relationships among variables. Principal component analysis (PCA) was performed to reduce data dimensionality and identify key influencing factors. Data visualization was carried out using GraphPad Prism 10 (GraphPad Software Inc., San Diego, CA, USA).

## 3 Results and analysis

### 3.1 Photosynthetic characteristics

**Figure 3:**
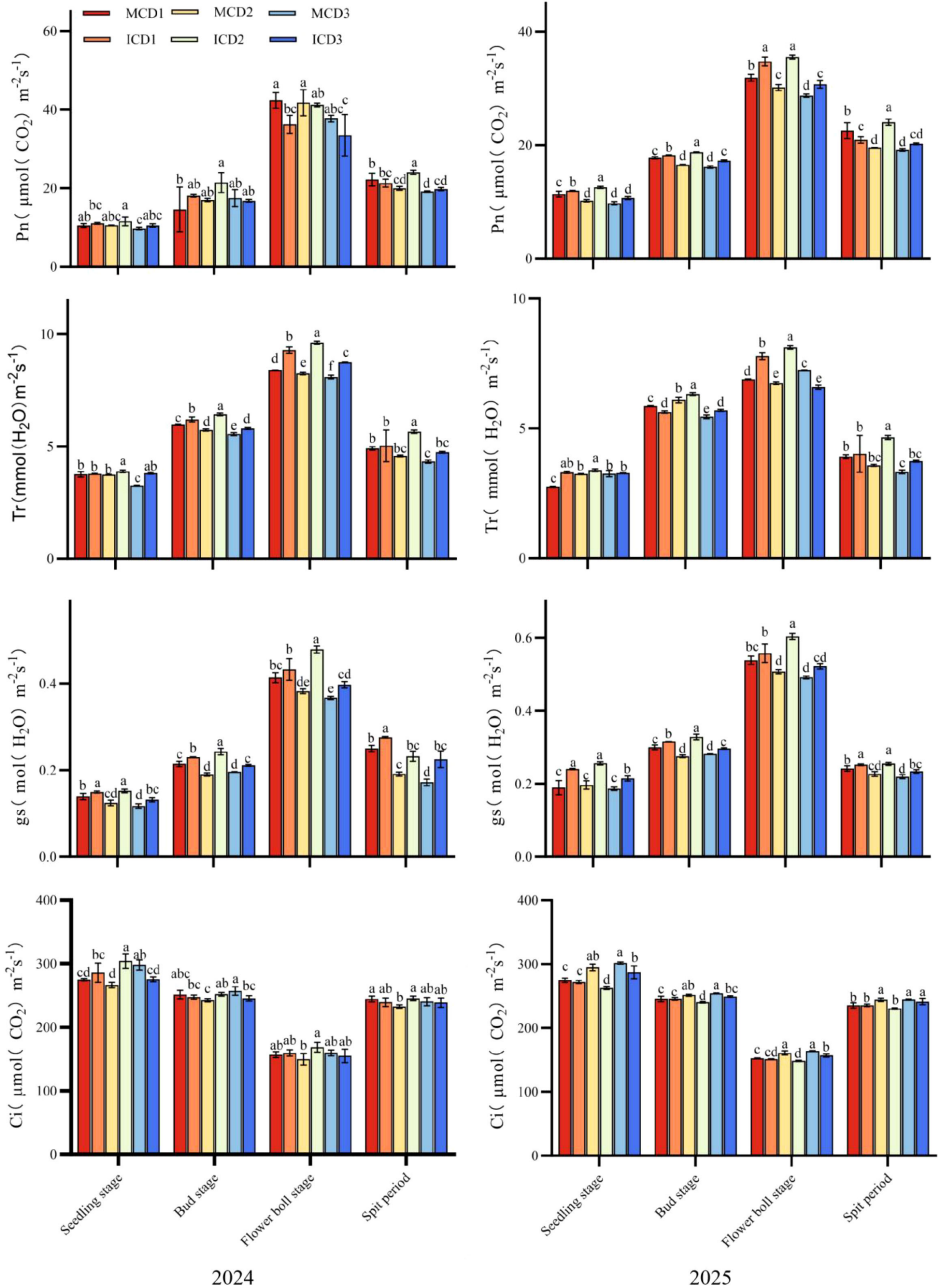
Effects of densification and peanut intercropping on cotton photosynthetic characteristics in 2024~2025.

As shown in **Figure 3**, the net photosynthetic rate (Pn), transpiration rate (Tr), and stomatal conductance (Gs) of cotton exhibited a unimodal trend over the growth period, increasing initially and then declining, with peak values observed at the flowering and boll-setting stage. In contrast, intercellular CO_2_ concentration (Ci) showed an opposite trend. Under monocropping conditions, Pn decreased with increasing planting density. At the flowering and boll-setting stage, MCD1 was significantly higher than MCD2 and MCD3 in both years. In contrast, under intercropping conditions, Pn was highest in the ICD2 treatment, which consistently outperformed ICD1 and ICD3 at this stage.

Comparing cropping patterns at the same density revealed that intercropping generally enhanced photosynthetic performance. For example, in 2025, the Pn of ICD2 was 8.4% higher than that of MCD2 at the flowering and boll-setting stage. Similar trends were observed for Tr and Gs. Under monocropping, both parameters decreased with increasing density, with MCD1 showing significantly higher values than MCD2 and MCD3. Under intercropping, ICD2 exhibited the highest Tr and Gs in both years. At D2 density, intercropped cotton showed increases of 10.1% and 15.6% in Gs compared with monocropped cotton in 2024 and 2025, respectively, while no significant difference was observed between the two cropping patterns at D1.

For Ci, monocropping treatments showed an increasing trend with planting density, particularly in 2025. At the flowering and boll-setting stage, Ci in MCD3 reached 175.42 μmol CO_2_ m^-2^ s^-^¹, which was 12.8% higher than that in MCD1. Under intercropping, ICD2 maintained relatively high Ci at the seedling and bud stages, whereas ICD1 exhibited the lowest Ci at the flowering and boll-setting stage.

### 3.2 Chlorophyll fluorescence parameters

**Figure 4:**
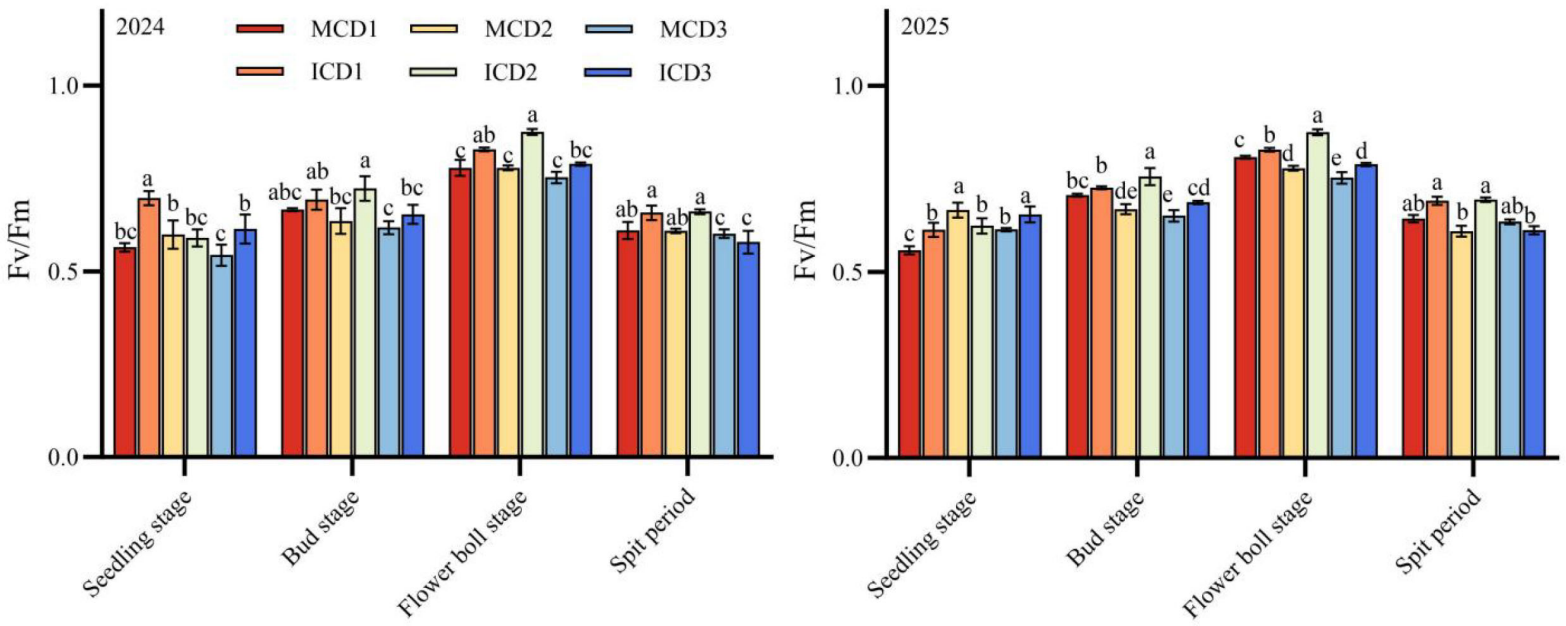
Effects of densification and peanut intercropping on psii photochemical efficiency (Fv/Fm) in cotton(2024-2025).

As shown in **Figure 4**, the maximum quantum efficiency of PSII (Fv/Fm) in cotton exhibited a unimodal trend during the growth period, increasing initially and then decreasing, with peak values observed at the flowering and boll-setting stage. Under monocropping conditions, Fv/Fm decreased with increasing planting density at this stage, following the order MCD1 > MCD2 > MCD3 in both 2024 and 2025. In contrast, under intercropping conditions, the ICD2 treatment showed the highest Fv/Fm, with significantly higher values than ICD1 and ICD3 in 2025.

Comparison between cropping patterns indicated that intercropping enhanced PSII efficiency, particularly at the D2 density level. At the flowering and boll-setting stage, Fv/Fm in ICD2 was 15.9% and 21.3% higher than that in MCD2 in 2024 and 2025, respectively.These results suggest that the D2 density configuration under intercropping is more favorable for maintaining higher PSII photochemical efficiency.

### 3.3 Effect of densification and intercropping of peanut on dynamic accumulation of dry matter in cotton

**Figure 5:**
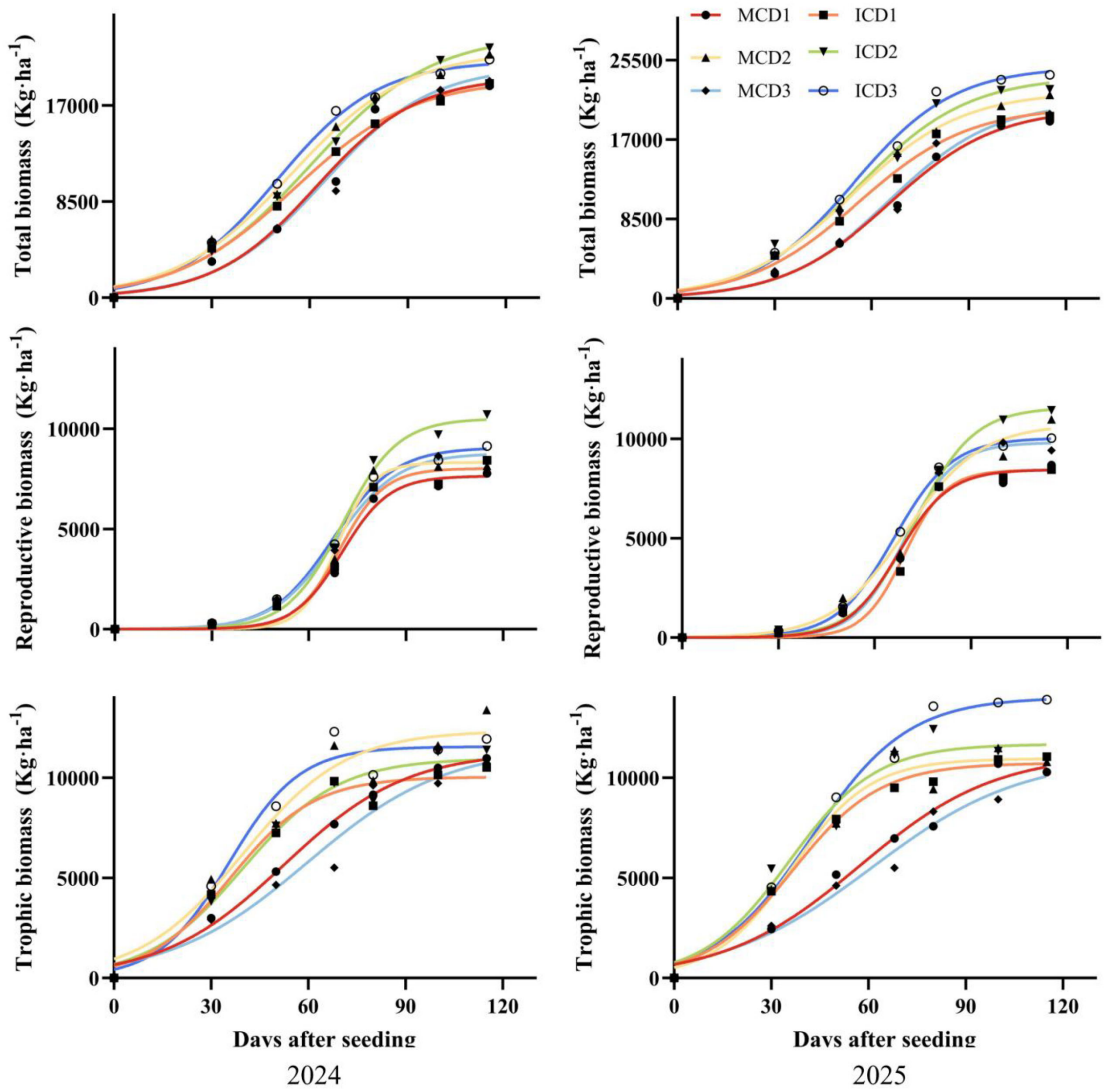
Effects of densification and peanut intercropping on cotton dry matter accumulation.

From 2024 to 2025, total biomass, reproductive biomass, and vegetative biomass of cotton under all treatments followed a typical sigmoid (“S-shaped”) growth pattern with increasing days after sowing, consistent with the logistic growth model (**Figure 5**). The coefficient of determination (R^2^) for all fitted models exceeded 0.97 (**Table 2**) (Equations 1–5), indicating a high goodness of fit. In terms of total dry matter accumulation, the ICD2 treatment achieved the highest biomass at 115 days after sowing in 2024, which was 8.9% higher than that of MCD1. In 2025, total biomass under MCD3 was 12.5% higher than that under MCD1. For reproductive biomass, ICD2 exhibited the highest accumulation in 2024, exceeding MCD1 and MCD2 by 32.9% and 21.6%, respectively. Regarding vegetative biomass, significant differences among treatments were observed in 2025, with MCD3 showing the highest value, which was 23.5% higher than that of MCD1.

**Table 2:** Characteristics of logistic growth model for cotton total dry matter accumulation under densification and peanut intercropping.

Year	Treatment	R^2^	V_m_ (kg·ha^-1^·d^-1^)	T_m_	T_1_	T_2_	ΔT	Gt (kg·ha^-1^)
				(d)				
2024	MCD1	0.9814	314.15	61.88	41.36	82.39	41.03	12888.69
	ICD1	0.9954	264.23	55.29	31.22	79.37	48.15	12723.04
	MCD2	0.9965	313.31	64.22	42.70	85.74	43.04	13484.22
	ICD2	0.9752	307.71	54.25	30.90	77.60	46.70	14370.06
	MCD3	0.9972	312.26	59.99	35.42	84.56	49.14	15344.76
	ICD3	0.9951	338.86	50.15	29.79	70.50	40.71	13794.84
2025	MCD1	0.9930	309.39	65.46	43.80	87.12	43.32	13403.28
	ICD1	0.9897	298.70	55.90	33.27	78.53	45.26	13518.08
	MCD2	0.9971	324.80	53.92	31.48	76.35	44.87	14574.14
	ICD2	0.9829	348.96	55.57	33.13	78.00	44.87	15657.90
	MCD3	0.9813	326.00	65.49	44.28	86.70	42.41	13827.07
	ICD3	0.9922	397.78	54.50	33.98	75.01	41.03	16319.42

T denotes the days after sowing; T_m_ represents the time when the maximum accumulation rate is reached; T_1_ marks the onset of rapid dry matter accumulation; T_2_ indicates the end of rapid dry matter accumulation; ΔT indicates the duration of rapid dry matter accumulation; V_m_ represents the maximum dry matter accumulation rate. Gt is the growth characteristic value, indicating that dry matter accumulation has reached 65.8% of the maximum accumulation.

Analysis of Logistic model parameters (**Table 2**) reveals that in 2024, the theoretical maximum accumulation (Gt) under MCD3 treatment was 11.2% and 19.1% higher than those under MCD2 and MCD1 treatments, respectively. The maximum accumulation rate (Vm) under ICD3 treatment was 8.5% higher than under MCD3. The rapid accumulation onset time (T_1_) showed that intercropping treatments generally preceded monoculture treatments, with ICD3 treatment in 2024 exhibiting a 9.84-day earlier T_1_ compared to MCD3. In 2025, ICD3 treatment demonstrated a 10.3-day earlier T_1_ than MCD3, indicating that intercropping promotes cotton’s early entry into the rapid growth phase.At the onset of cotton’s rapid dry matter accumulation, intercropped peanuts were still at the seedling to early flowering stage with small canopy and no significant shading. Weak interspecific competition and positive effects such as biological nitrogen fixation jointly promoted earlier rapid growth in cotton. Regarding growth characteristics (Gt), the MCD3 treatment in 2024 recorded the highest value, 19.1% higher than MCD1, while the ICD3 treatment in 2025 showed a 21.7% higher Gt compared to MCD1.

### 3.5 Effects of increasing density and intercropping peanuts on cotton yield

As shown in **Table 3**, cotton yield in monoculture systems decreased with increasing planting density. In 2024, MCD1’s seed cotton yield was 4.6% and 11.5% higher than MCD2 and MCD3, respectively. By 2025, the yield gap widened to 5.1% and 14.4% for MCD1 compared to MCD2 and MCD3. The Density Contribution Rate (RCTD) (Equation 6) further confirmed this trend, with both D2 and D3 showing negative RCTD values under monoculture conditions, indicating that increased planting density negatively impacted cotton yield.

**Table 3:** Effects of plant density and intercropping on cotton yield in cotton–peanut systems.

Year	Cropping patterns	RCTD(%)		Cotton(kg)		Peanut (kg·ha^-^¹)	LER
		MC	IC	MC	IC		
2024	MP	——		——	——	7158.85 ± 65.65 a	——
	D1	——		7065.29 ± 52.82 a	4462.82 ± 45.23 b	3315.83 ± 44.01 b	1.10 ± 0.007 b
	D2	-4.37	12.07	6756.69 ± 44.29 b	5001.7 ± 31.98 a	3388.38 ± 12.05 b	1.21 ± 0.003 a
	D3	-10.32	2.24	6336.13 ± 47.09 c	4562.69 ± 70.34 b	3150.74 ± 69.94 c	1.15 ± 0.017 b
2025	MP	——		——	——	7200.48 ± 65.72 a	——
	D1	——		7320.71 ± 101.35 a	4557.05 ± 34.25 c	3590.24 ± 42.47 b	1.12 ± 0.004 c
	D2	-4.86	13.66	6964.91 ± 85.61 b	5179.64 ± 27.32 a	3513.39 ± 48.74 b	1.23 ± 0.020 a
	D3	-12.60	3.54	6398.52 ± 45.33 c	4718.26 ± 35.29 b	3278.98 ± 38.32 c	1.19 ± 0.005 b

RCTD (Density Contribution Rate) is calculated and compared under different planting patterns, with the value under D1 density treatment serving as the baseline.

Under intercropping conditions, cotton yield exhibited a trend of initially increasing and then decreasing with density, with D2 density yielding the optimal results. In 2024, the lint yield of ICD2 was 12.1% and 8.7% higher than that of ICD1 and ICD3, respectively. In 2025, the yield of ICD2 was 12.0% and 8.7% higher than that of ICD1 and ICD3, respectively, with both differences reaching significant levels. Regarding RCTD, both D2 and D3 densities yielded positive values under intercropping, with D2’s RCTD exceeding D3’s. This indicated that moderate densification in intercropping systems could enhance yields, though excessive density still led to reductions. At identical densities, intercropped cotton yields surpassed those of monocropping significantly. Peanut yields in intercropping systems decreased with increasing cotton density. Peanut yield under the D1 density in the intercropping system was significantly higher than that under D3 density in both 2024 and 2025. The LER (Equation 7) was greater than 1 for all intercropping treatments, indicating a clear yield advantage for cotton-peanut intercropping. The highest LER was observed at D2 density, reaching 1.21 in 2024 and 1.23 in 2025, significantly higher than the D1 treatment. This suggested that the medium-density intercropping system achieved optimal land use efficiency.

### 3.6 Correlation analysis

**Figure 6:**
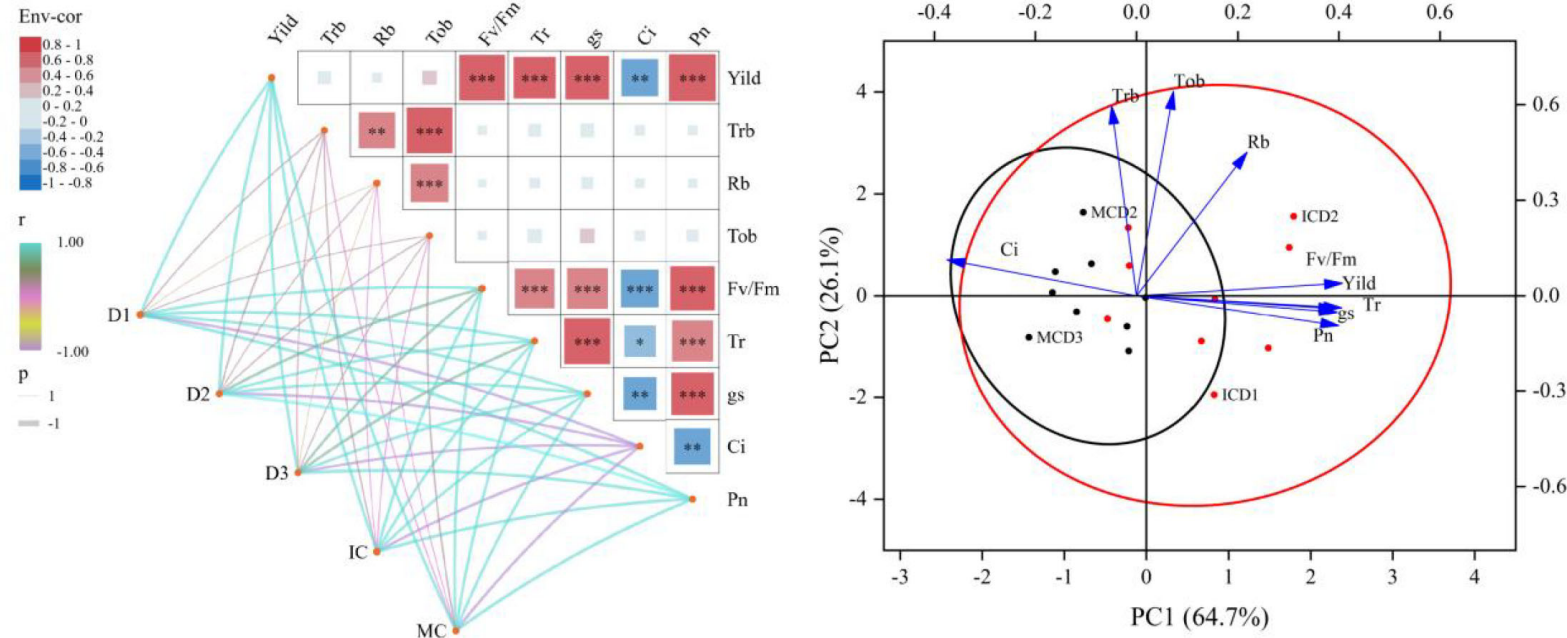
Correlation analysis and principal of cotton indicators in response to planting density and cotton-peanut intercropping. Pn, net photosynthetic rate; Ci, intercellular CO_2_ concentration; gs, stomatal conductance; Tr, transpiration rate; Fv/Fm, maximum photochemical efficiency of PSII; Tob, total dry matter accumulation; Rb, reproductive organ dry matter accumulation; Trb, vegetative organ dry matter accumulation; Yield, seed cotton yield.Nodes represent treatment factors (D1/D2/D3, planting densities; IC, intercropping; MC, monocropping). Lines represent Pearson correlation coefficients between treatments and indicators, with color (cyan=positive, purple/yellow=negative, yellow-green=weak/no correlation) and thickness indicating correlation direction and magnitude. Significant differences were determined at the P<0.05 level. This correlation analysis was based on data collected at the flowering and boll-setting stage during 2024~2025.

As shown in **Figure 6**, cotton yield exhibited highly significant positive correlations with the maximum quantum efficiency of PSII (Fv/Fm), net photosynthetic rate (Pn), and stomatal conductance (Gs). In contrast, intercellular CO_2_ concentration (Ci) was significantly negatively correlated with Pn, Gs, and Tr. Fv/Fm showed highly significant positive correlations with Pn, Gs, and Tr, and a highly significant negative correlation with Ci, indicating a close association between PSII photochemical efficiency and photosynthetic gas exchange parameters.

Dry matter accumulation in vegetative organs (Trb) was significantly positively correlated with that in reproductive organs (Rb), which in turn showed a highly significant positive correlation with total dry matter accumulation (Tob). In addition, Trb exhibited a highly significant positive correlation with Fv/Fm.

Planting density and cropping pattern also influenced the overall relationships among variables. The D2 density and intercropping pattern (IC) were positively associated with yield and photosynthetic parameters, suggesting that moderate densification combined with intercropping enhances photosynthetic performance and biomass accumulation.

Principal component analysis (PCA) showed that the first two principal components explained 90.8% of the total variance, with PC1 and PC2 accounting for 64.7% and 26.1%, respectively. PC1 was characterized by strong positive loadings of Pn, Gs, Tr, Fv/Fm, and yield, along with a negative loading of Ci, representing the photosynthetic productivity dimension. PC2 was mainly associated with dry matter accumulation variables, including Tob, Trb, and Rb, representing the biomass accumulation dimension.

The distribution of treatments in the PCA space showed clear differentiation. ICD2 was located in the first quadrant, indicating high photosynthetic capacity and biomass accumulation. MCD2 was distributed in the intermediate region, whereas MCD3 was located in the third quadrant, reflecting relatively lower photosynthetic performance. ICD1 was positioned in the fourth quadrant, showing relatively strong photosynthetic capacity but lower biomass accumulation.

Overall, ICD2 exhibited superior performance in terms of photosynthetic characteristics, dry matter accumulation, and yield formation.

## 4 Discussion

### 4.1 Effects of densification and intercropping on cotton yield and dry matter accumulation

Intercropping achieves efficient resource utilization through ecological niche complementarity among crops, serving as an effective approach to enhance land productivity (Feng et al., 2021).This study found that over the two-year period, the cotton yield of the ICD2 system was significantly higher than that of both the ICD1 and ICD3 systems. Furthermore, the Land Equivalent Ratio (LER) for all intercropping treatments exceeded 1 (**Table 3**). These results are consistent with previous research indicating that cotton intercropping systems offer yield advantages. These findings align with previous research conclusions indicating yield advantages in cotton intercropping systems (Yang et al., 2025).

The spatial and temporal complementarity between cotton and peanut in light, heat, water, and nutrient utilization results in weaker interspecific competition than intraspecific competition, facilitating efficient resource utilization (Chi et al., 2019). Additionally, peanut’s biological nitrogen fixation improves soil nitrogen supply, indirectly promoting cotton growth and development (Tang et al., 2020; Wang et al., 2022). Furthermore, the marginal row dominance effect in intercropping systems amplifies cotton’s access to ample sunlight and growth space, thereby increasing biomass accumulation (Wu et al., 2026).

All cotton treatments demonstrated dry matter accumulation consistent with the Logistic growth model (**Table 2**). The intercropping treatments showed earlier onset of rapid accumulation (T_1_) compared to monoculture treatments. Specifically, in 2024, ICD3 achieved a 5.57-day earlier peak than MCD3, indicating that intercropping accelerates cotton’s entry into the rapid growth phase.

The ICD2 treatment recorded 20.0% higher total dry matter accumulation than MCD1 (**Figure 5**), with reproductive organ dry matter accumulation exceeding MCD1 by 42.9% and MCD2 by 31.6%. This increased dry matter accumulation suggests enhanced photosynthetic product supply, while the higher reproductive organ allocation reflects optimized harvest index. These synergistic effects collectively contributed to improved seed cotton yield.

In monoculture systems, cotton yield decreases with increasing planting density, whereas intercropping effectively mitigates the adverse effects of density increase (**Table 3**), demonstrating greater tolerance to density enhancement. The yield reduction under monoculture may result from intensified intra-population competition at high densities, where plants compete more intensely for light, water, and nutrients, limiting individual growth (Chapepa et al., 2020). Additionally, excessive density causes canopy closure, severely shading lower leaves and reducing effective photosynthetic radiation interception, thereby impairing photosynthetic product synthesis and accumulation (Zhai et al., 2024).

In intercropping systems, the height difference between cotton and peanuts forms a three-dimensional composite population structure, providing better canopy ventilation and light transmission than monoculture, thus enhancing adaptability to density increase. However, when density was further increased to D3 (300, 000 plants·ha^-^¹), the yield of ICD3 treatment remained lower than ICD2 for two consecutive years.

The excessively high density D3 exceeds the resource carrying threshold of the intercropping system, intensifying both intra-species and inter-species competition, completely negating the ecological complementary advantages of cotton plants. The dense canopy structure disrupts the stratification of light, leading to significant declines in photosynthesis and PSII efficiency; rapid accumulation and early decline of dry matter, reduced reproductive allocation efficiency; the competition of cotton plants shifts from complementarity to antagonism, the nitrogen fixation supply advantage is lost, both LER and RCTD significantly decrease, and the superiority of the densified population cannot compensate for the decline in individual plant traits, ultimately resulting in the loss of the intercropping high-yield advantage (Dong et al., 2024). Therefore, appropriate density configuration is crucial for maximizing the yield-enhancing advantages of intercropping.

### 4.2 Effects of densification and intercropping on cotton photosynthetic characteristics and their relationship with yield

Photosynthesis serves as the physiological foundation for crop dry matter accumulation and yield formation, with over 90% of cotton yield derived from photosynthetic products after the flowering and boll-setting stage (Mahato, 2024). This study revealed a highly significant positive correlation between yield and net photosynthetic rate (Pn) and maximum photosystem II (PSII) photochemical efficiency (Fv/Fm) during the flowering and boll-setting stage (**Figure 6**), confirming that improved photosynthetic performance is the direct driver of yield increase.

The ICD2 treatment demonstrated optimal Pn, Gs, and Tr values during the flowering and boll-setting stage (**Figure 3**). In 2025, intercropped cotton under D2 density achieved a 15.6% higher Gs compared to monoculture. The increased stomatal conductance indicates enhanced stomatal opening, facilitating CO_2_ uptake for photosynthesis while promoting transpiration to maintain optimal leaf temperature and water metabolism (Leng et al., 2024). The elevated Pn reflects enhanced carbon assimilation capacity (Fan et al., 2025), consistent with the trend of Gs, suggesting that the improvement in cotton photosynthetic capacity under intercropping and densification was primarily regulated by non-stomatal factors.

Additionally, under monoculture conditions, Ci increased with density, with the high-density treatment MCD3 showing significantly higher Ci than MCD1, likely due to reduced leaf photosynthetic rate and CO_2_ consumption under high density. Fv/Fm, which reflects the photoenergy conversion efficiency of PSII reaction centers, is a critical indicator for evaluating the functional status of plant photosynthetic apparatus (Muhammad et al., 2021). The ICD2 treatment exhibited significantly higher Fv/Fm (**Figure 4**), with a 21.3% increase in 2025 compared to MCD2, indicating that intercropping combined with appropriate density effectively maintains efficient PSII reaction center operation and reduces photoinhibition.

This may be attributed to the intercropping system’s improvement of the cotton canopy microenvironment, including temperature and humidity regulation as well as optimized light distribution, thereby preserving the integrity and functional activity of photosynthetic organs (Li et al., 2023). ICD2 treatment demonstrated optimal performance on both PC1 and PC2 (**Figure 6**), exhibiting higher photosynthetic capacity and dry matter accumulation, thus achieving synergistic yield enhancement.

## 5 Conclusion

This study demonstrates that under the arid conditions of southern Xinjiang, the cotton–peanut intercropping system with a planting density of 240, 000 plants·ha^-1^ (D2) is optimal for improving cotton productivity and resource-use efficiency. At the flowering and boll-setting stage, the ICD2 treatment exhibited superior photosynthetic performance (Pn, Gs, and Tr) and PSII efficiency (Fv/Fm), with Gs and Fv/Fm increasing by 12.85% and 21.3%, respectively, compared with monocropping at the same density. Although cotton yield under monocropping declined with increasing density, intercropping effectively mitigated this negative effect, with ICD2 achieving the highest yield in both years and a land equivalent ratio (LER) of 1.23. Correlation and principal component analyses further confirmed that yield improvement was closely associated with enhanced photosynthetic capacity and PSII efficiency, with ICD2 identified as the optimal treatment.

Overall, moderate densification combined with cotton–peanut intercropping enhances photosynthetic performance, promotes efficient biomass accumulation, and increases yield. These findings provide both theoretical support and practical guidance for alleviating the grain–cotton land-use conflict and optimizing cotton production systems in arid regions.

## Data Availability

The raw data supporting the conclusions of this article will be made available by the authors, without undue reservation.
